# Supplementary material for: Stochastic colonization of hosts with a finite lifespan can drive individual host microbes out of equilibrium
Source: PLoS Comput Biol. 2020 Nov 2;16(11):e1008392. doi: 10.1371/journal.pcbi.1008392 (PMC7660904; doi:10.1371/journal.pcbi.1008392)
Supplement: S1 Appendix — (PDF) [file pcbi.1008392.s001.pdf]

Stochastic colonization of hosts with a finite lifespan can drive individual host microbes out of equilibrium

Román Zapién-Campos[1], Michael Sieber[1], Arne Traulsen[1,*]

1 Max Planck Institute for Evolutionary Biology, August-Thienemann-Str. 2, 24306 Plön, Germany

* traulsen@evolbio.mpg.de

Supporting Information

S1 Appendix

Derivation of the Fokker-Planck approximation with resetting

Define a Markov process in the discrete state space $\{0, 1, ..., N\}$, where N is the maximum capacity for microbes within a single host. Let n_i be the number of individuals of the *i-th* microbial taxon for $i = \{1, ..., M\}$ and n_0 the amount of unoccupied space. The probability of being in state n_i at time $t + 1$ is given by the sum of the probabilities to go in, out and remain in n_i at time t,

$$\Phi_i[n_i, t+1] = \sum_{o_i=0}^{N} P_i[o_i \to n_i]\Phi_i[o_i, t],$$

where $\Phi_i[o_i, t]$ is the probability of being in state o_i at time t, and $P_i[o_i \to n_i]$ is the probability of transitioning from state o_i to n_i. Taking out the probability of remaining at n_i we get

$$\Phi_i[n_i, t+1] = P_i[n_i \to n_i]\Phi_i[n_i, t] + \sum_{o_i \neq n_i} P_i[o_i \to n_i]\Phi_i[o_i, t]$$

However $P_i[n_i \to n_i] = 1 - \sum_{o_i \neq n_i} P_i[n_i \to o_i]$. Therefore

$$\Phi_i[n_i, t+1] - \Phi_i[n_i, t] = -\sum_{o_i \neq n_i} P_i[n_i \to o_i]\Phi_i[n_i, t] + \sum_{o_i \neq n_i} P_i[o_i \to n_i]\Phi_i[o_i, t]$$

Dividing by Δt and taking the continuous time limit, we find the time continuous master equation

$$\frac{\partial \Phi_i[n_i, t]}{\partial t} = -\sum_{o_i \neq n_i} T_i[n_i \to o_i]\Phi_i[n_i, t] + \sum_{o_i \neq n_i} T_i[o_i \to n_i]\Phi_i[o_i, t],$$

where $T_i[n_i \to o_i]$ and $T_i[o_i \to n_i]$ are transition rates. This equation contains transitions to and from neighbouring states of n_i, i.e. $n_i - 1$ and $n_i + 1$, but also to and from non-neighbouring states due to resetting events.

The next derivation steps focus on approximating the neighbouring transitions locally around n_i. However, because the resetting events are non-neighbouring transitions, we have to treat them separately. To do this, we assume that each

$T_i[n_i \to o_i]$ and $T_i[o_i \to n_i]$ can be linearly separated in contributions due to resetting and non-resetting events.

Define R_i as the only state towards which the resetting occurs, so the master equation for this state is

$$\frac{\partial \Phi_i[R_i, t]}{\partial t} = -\sum_{o_i \neq R_i} T_i[R_i \to o_i]\Phi_i[R_i, t] + \sum_{o_i \neq R_i} T_i^*[o_i \to R_i]\Phi_i[o_i, t] + \sum_{o_i \neq R_i} \tau \Phi_i[o_i, t],$$

where $T_i^*[o_i \to R_i]$ are transition rates from neighbouring o_i to R_i, and τ is the transition rate from non-neighbouring o_i to R_i (which we assume to be independent of o_i). We can rewrite this as

$$\frac{\partial \Phi_i[R_i, t]}{\partial t} = -\sum_{o_i \neq R_i} T_i[R_i \to o_i]\Phi_i[R_i, t] + \sum_{o_i \neq R_i} T_i^*[o_i \to R_i]\Phi_i[o_i, t] + \tau(1 - \Phi_i[R_i, t])$$

For any other state $n_i^* := n_i \neq R_i$, the master equation is

$$\frac{\partial \Phi_i[n_i^*, t]}{\partial t} = -\sum_{o_i \neq n_i^*} T_i^*[n_i^* \to o_i]\Phi_i[n_i^*, t] - \tau \Phi_i[n_i^*, t] + \sum_{o_i \neq n_i^*} T_i[o_i \to n_i^*]\Phi_i[o_i, t],$$

where $T_i^*[n_i^* \to o_i]$ are transition rates from n_i^* to neighbouring o_i. We can combine both master equations in the following way

$$\frac{\partial \Phi_i[n_i, t]}{\partial t} = -\sum_{o_i \neq n_i} T_i^*[n_i \to o_i]\Phi_i[n_i, t] + \sum_{o_i \neq n_i} T_i^*[o_i \to n_i]\Phi_i[o_i, t] + \tau\left(\delta_{n_i, R_i} - \Phi_i[n_i, t]\right),$$

where δ_{n_i, R_i} is the Kronecker delta (1 for $n_i = R_i$ and 0 in all other cases), and $\tau \Phi_i[n_i, t]$ and $\tau \delta_{n_i, R_i}$ are the non-neighbouring outflux and influx, respectively.

Now we can approximate the neighbouring transitions contained in the sums. We define $x_i = n_i/N$ and $r_i = R_i/N$, which are approximately continuous in the large N limit. With this the sums are replaced by integrals

$$\begin{aligned}\frac{\partial \Phi_i[x_i, t]}{\partial t} = &-\int T_i^*[x_i \to x_i + \Delta x_i]\Phi_i[x_i, t] d\Delta x_i + \int T_i^*[x_i + \Delta x_i \to x_i]\Phi_i[x_i + \Delta x_i, t] d\Delta x_i \\ &+ \tau\left(\delta_{x_i, r_i} - \Phi_i[x_i, t]\right),\end{aligned}$$

where δ_{x_i, r_i} is 1 for $x_i = r_i$ and 0 otherwise. We focus on the interval Δx_i around x_i to obtain the Taylor expansion of the influx, $\int T_i^*[x_i + \Delta x_i \to x_i]\Phi_i[x_i + \Delta x_i, t] d\Delta x_i$

$$\begin{aligned}= &\int T_i^*[x_i \to x_i - \Delta x_i]\Phi_i[x_i, t] d\Delta x_i - \int \Delta x_i \frac{\partial}{\partial x_i}(T_i^*[x_i \to x_i - \Delta x_i]\Phi_i[x_i, t]) d\Delta x_i + \\ &\int \frac{(\Delta x_i)^2}{2} \frac{\partial^2}{\partial x_i^2}(T_i^*[x_i \to x_i - \Delta x_i]\Phi_i[x_i, t]) d\Delta x_i + ...\end{aligned}$$

Realizing that the magnitude of the zeroth order term equals the outflux, truncating the expansion at the second order and putting terms together, we find

$$\begin{aligned}\frac{\partial \Phi_i[x_i, t]}{\partial t} = &-\frac{\partial}{\partial x_i}\left(\Phi_i[x_i, t] \int \Delta x_i T_i^*[x_i \to x_i - \Delta x_i] d\Delta x_i\right) \\ &+ \frac{1}{2}\frac{\partial^2}{\partial x_i^2}\left(\Phi_i[x_i, t] \int (\Delta x_i)^2 T_i^*[x_i \to x_i - \Delta x_i] d\Delta x_i\right) + \tau\left(\delta_{x_i, r_i} - \Phi_i[x_i, t]\right)\end{aligned}$$

Which simplifies to

$$\boxed{\frac{\partial}{\partial t}\Phi_i[x_i,t] = \frac{\partial}{\partial x_i}\left(-a_i[x_i]\Phi_i[x_i,t] + \frac{1}{2}\frac{\partial}{\partial x_i}\left(b_i^2[x_i]\Phi_i[x_i,t]\right)\right) + \tau\left(\delta_{x_i,r_i} - \Phi_i[x_i,t]\right),} \tag{S1}$$

where $a_i[x_i]$ and $b_i^2[x_i]$ are the expected change and squared-change of x_i, respectively. These quantities are derived in the main text based on the process of death, birth and immigration of microbes. Although, the change of x_i depends on all $k \neq i$. Derived from our assumptions, $a_i[x_i]$ and $b_i^2[x_i]$ contain all the information of a host microbiome from the *j-th* microbial taxon or unoccupied space ($i = 0$) perspective.

Importantly, host death, expressed by $\tau(\delta_{x_i,r_i} - \Phi_i[x_i,t])$, has opposite effects on microbes and unoccupied space. For a microbial taxon the frequency resets to zero, $r_i = 0$, while for unoccupied space it resets to one, $r_0 = 1$. Eq (S1) is the same as Eq (2) in the main text.

A numerical solution of the stationary model, Eq (5) in the main text, can be found using the boundary conditions $\frac{d\Phi_0[0]}{dx_0} = \frac{d\Phi_0[1]}{dx_0} = 0$ and $\frac{d\Phi_i[0]}{dx_i} = \frac{d\Phi_i[1]}{dx_i} = 0$, alongside $\Phi_0[1] = 1$ for unoccupied space and $\Phi_i[0] = 1$ for microbes [1], enforcing the normalization condition

$$\int_0^1 \Phi_i[x_i]dx_i = 1$$

References

1. Kumar P, Narayanan S. Solution of Fokker-Planck equation by finite element and finite difference methods for nonlinear systems. Sadhana. 2006;31(4):445–461.
