# Supplementary figures and images for: Stochastic colonization of hosts with a finite lifespan can drive individual host microbes out of equilibrium
Source: PLoS Comput Biol. 2020 Nov 2;16(11):e1008392. doi: 10.1371/journal.pcbi.1008392 (PMC7660904; doi:10.1371/journal.pcbi.1008392)

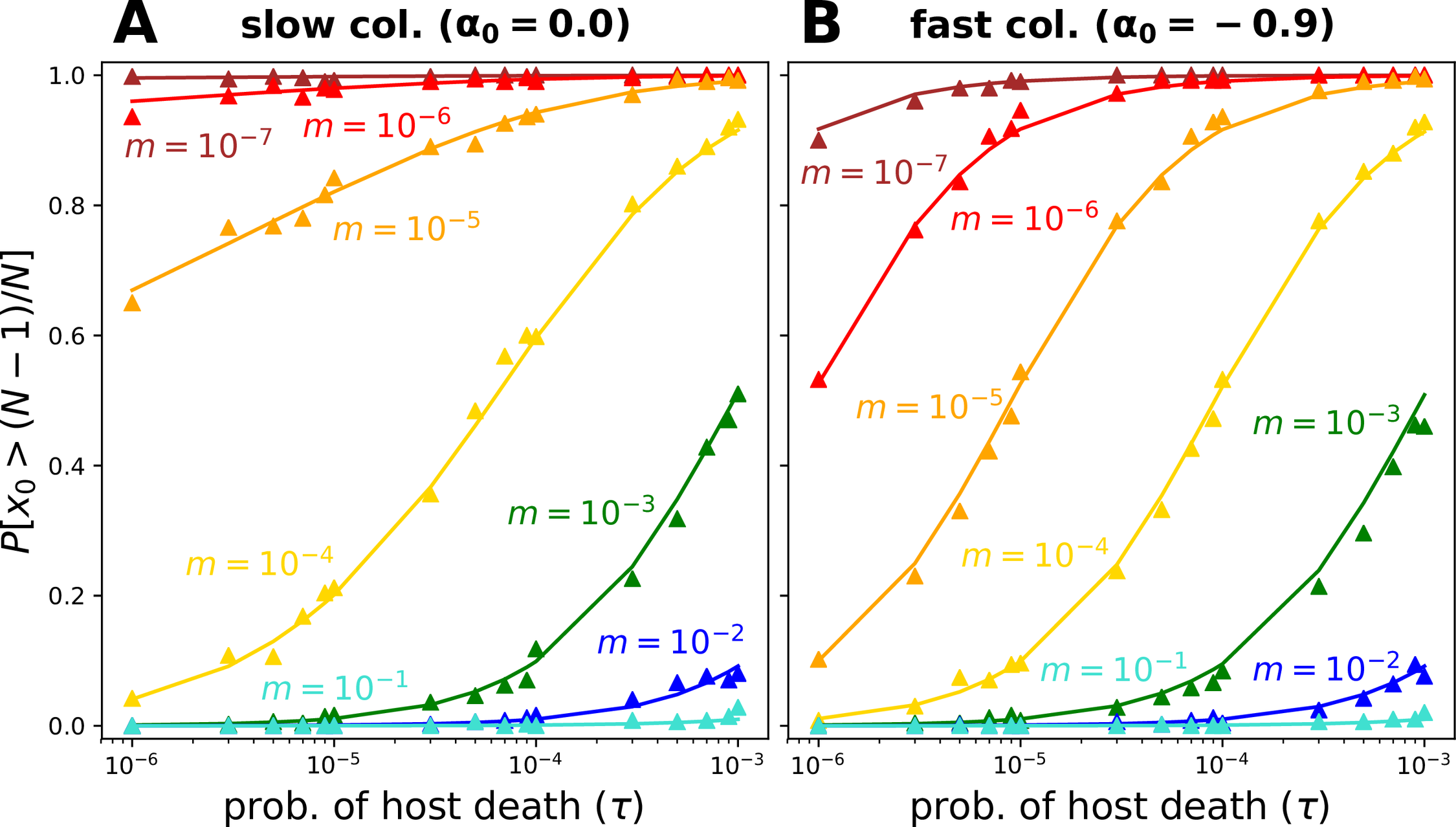

Supplement: S1 Fig — The P[x0 > (N − 1)/N] is shown, Eq (10). Lines show the model prediction, while triangles show the average over the steady state of 500 host samples according to Eq (6). The match spans several magnitude orders of migration (m) and probability of host death-birth events (τ). The probability increases for shorter host lifespans (larger τ) and less migration to the hosts (smaller m). The rate of occupation of empty space (α0) has a larger effect on cases where migration is limited and the host lifespan is long (small τ). Simulations were computed as explained in the Methods. Other parameters: N = 104. We use Eq (5a) where no definition of pi and αi is required. (TIF) [file pcbi.1008392.s002.tif]

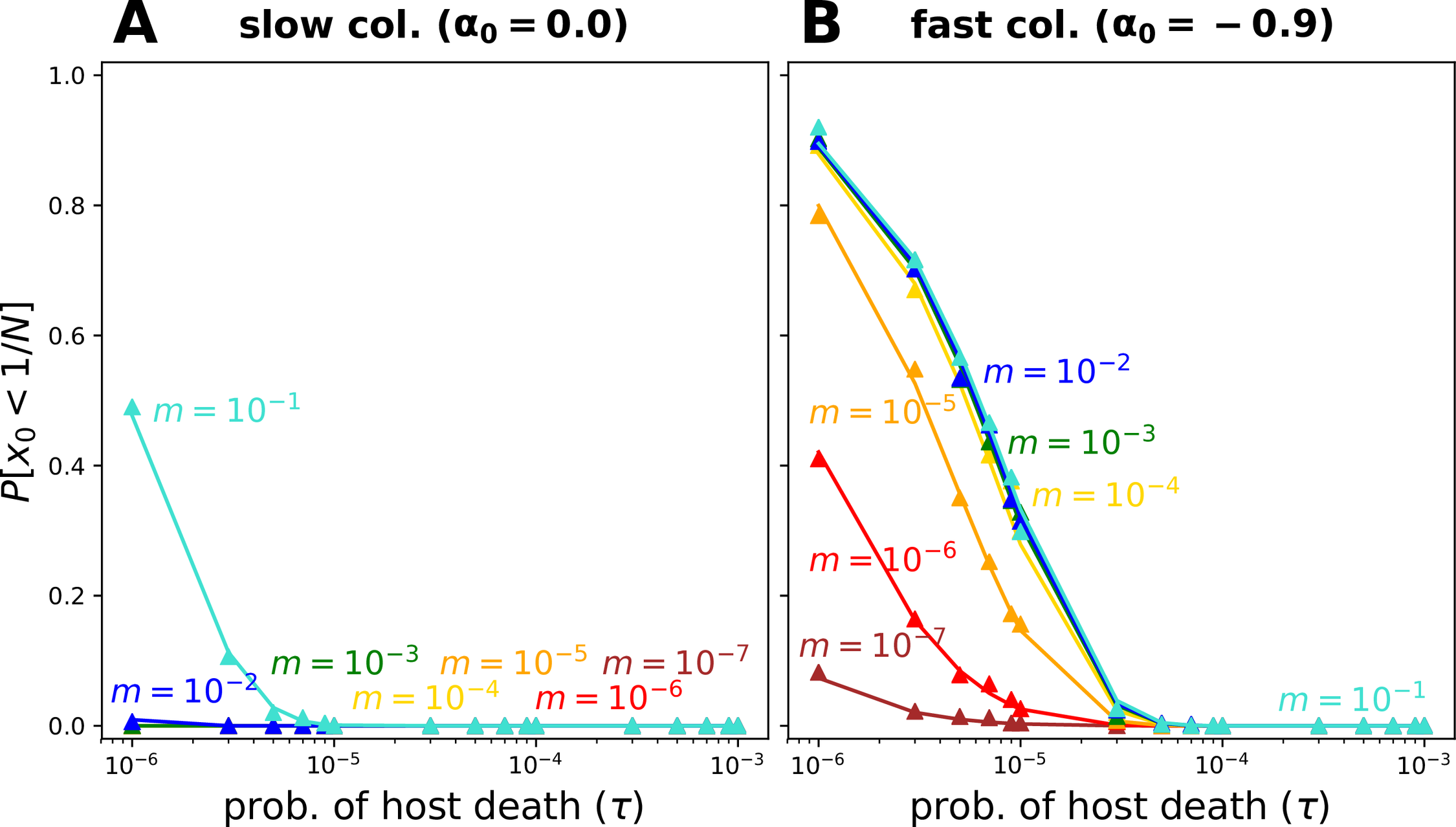

Supplement: S2 Fig — The P[x0 < 1/N] is shown, Eq (9). Lines show the model prediction, while triangles show the average over the steady state of 500 host samples according to Eq (6). The match spans several magnitude orders of migration (m) and probability of host death-birth events (τ). The probability increases for longer host lifespans (smaller τ) and larger migration to the hosts (larger m). The rate of occupation of empty space (α0) has a larger effect on cases where migration is large and the host lifespan is long (small τ). Simulations were computed as explained in the Methods. Other parameters: N = 104. We use Eq (5a) where no definition of pi and αi is required. (TIF) [file pcbi.1008392.s003.tif]

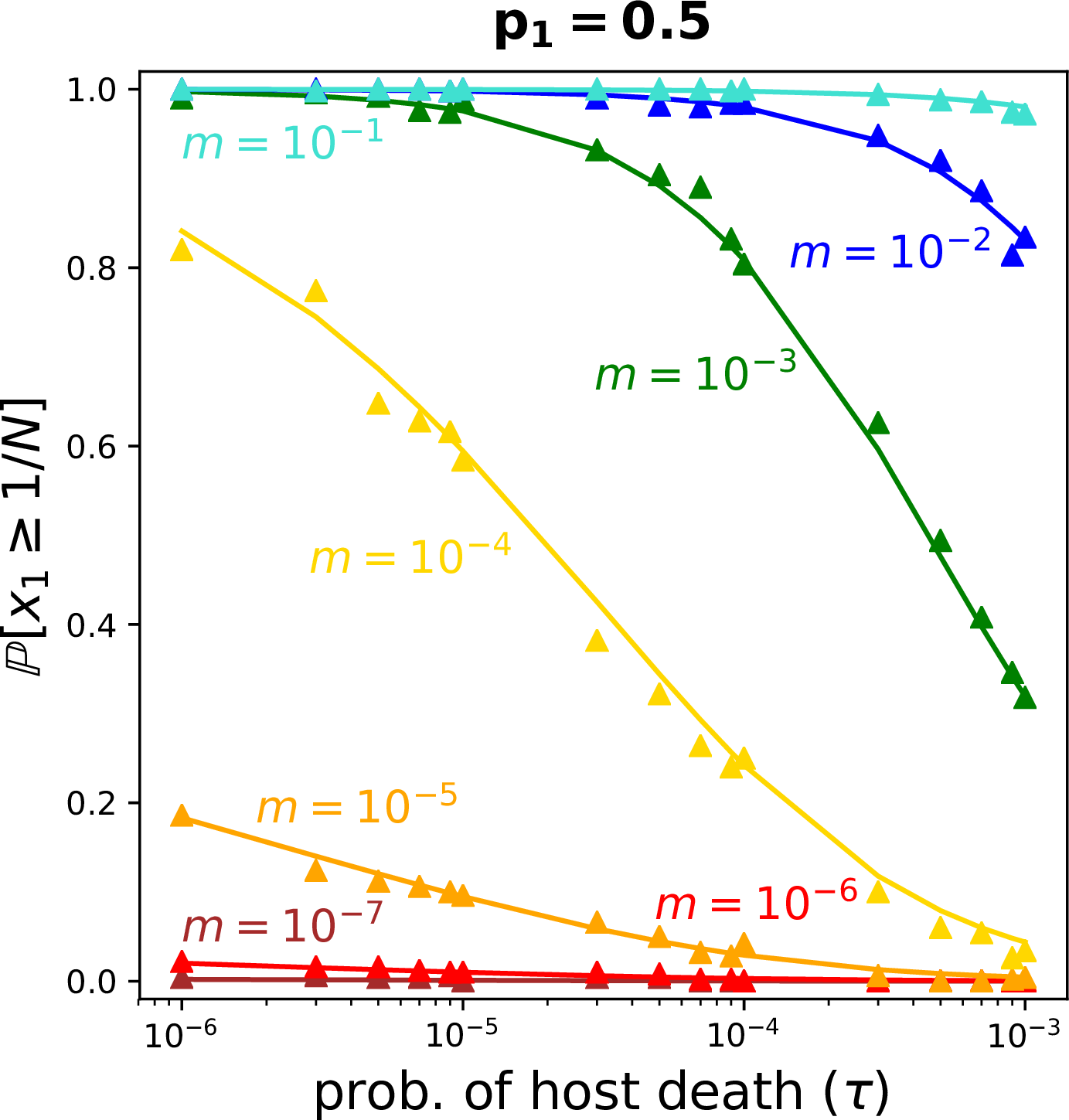

Supplement: S3 Fig — p1 indicates the frequency of microbial taxon 1 in the pool of colonizers. Lines show the model prediction, while triangles show the average over the steady state of 500 host samples according to Eq (6). The match spans several magnitude orders of migration (m) and probability of host death-birth events (τ). The probability increases for longer host lifespans (smaller τ) and larger migration to the hosts (larger m). Simulations were computed as explained in the Methods. Other parameters: N = 104 and α0 = α1 = 0. (TIF) [file pcbi.1008392.s004.tif]

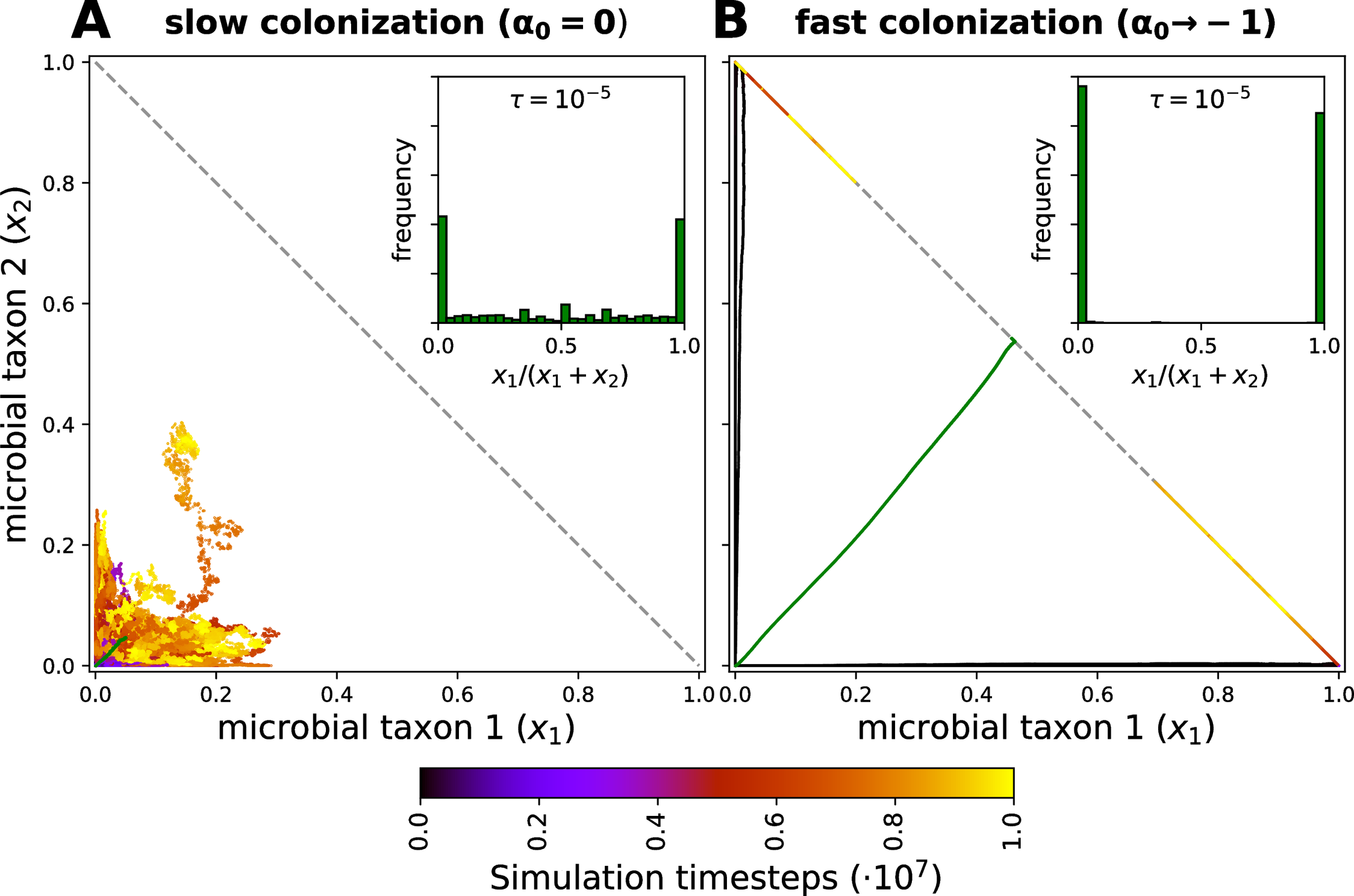

Supplement: S4 Fig — Except for m = 10−4, all parameters are equal to those in Fig 2. (A) The limited migration and slow empty space occupation impedes the host from being colonized completely. (B) When empty space is occupied rapidly, although the mean is conserved, the distribution becomes sharply bimodal as a result of the fast proliferation of the first colonizer, and a slow convergence to the long-term equilibrium, which within the time-range simulated is not reached. For finite host lifespans, such dynamics can produce alternative microbiomes and partial colonization of hosts in the equilibrium. (TIF) [file pcbi.1008392.s005.tif]

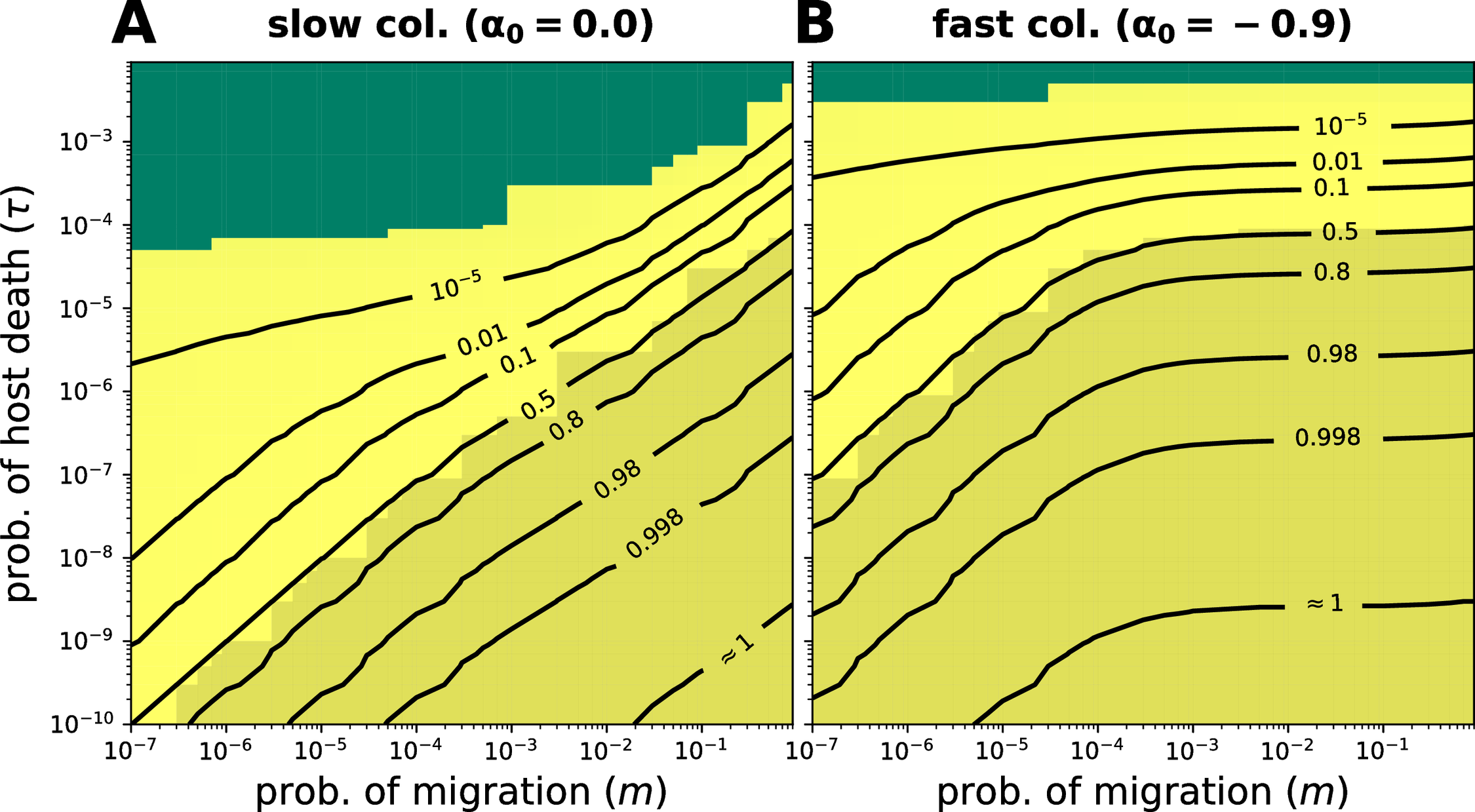

Supplement: S5 Fig — Except for N = 103, all parameters are equal to those in Fig 5. The smaller capacity for microbes of a host makes full colonization more likely, and migration (m) has increased influence for larger τ. (TIF) [file pcbi.1008392.s006.tif]

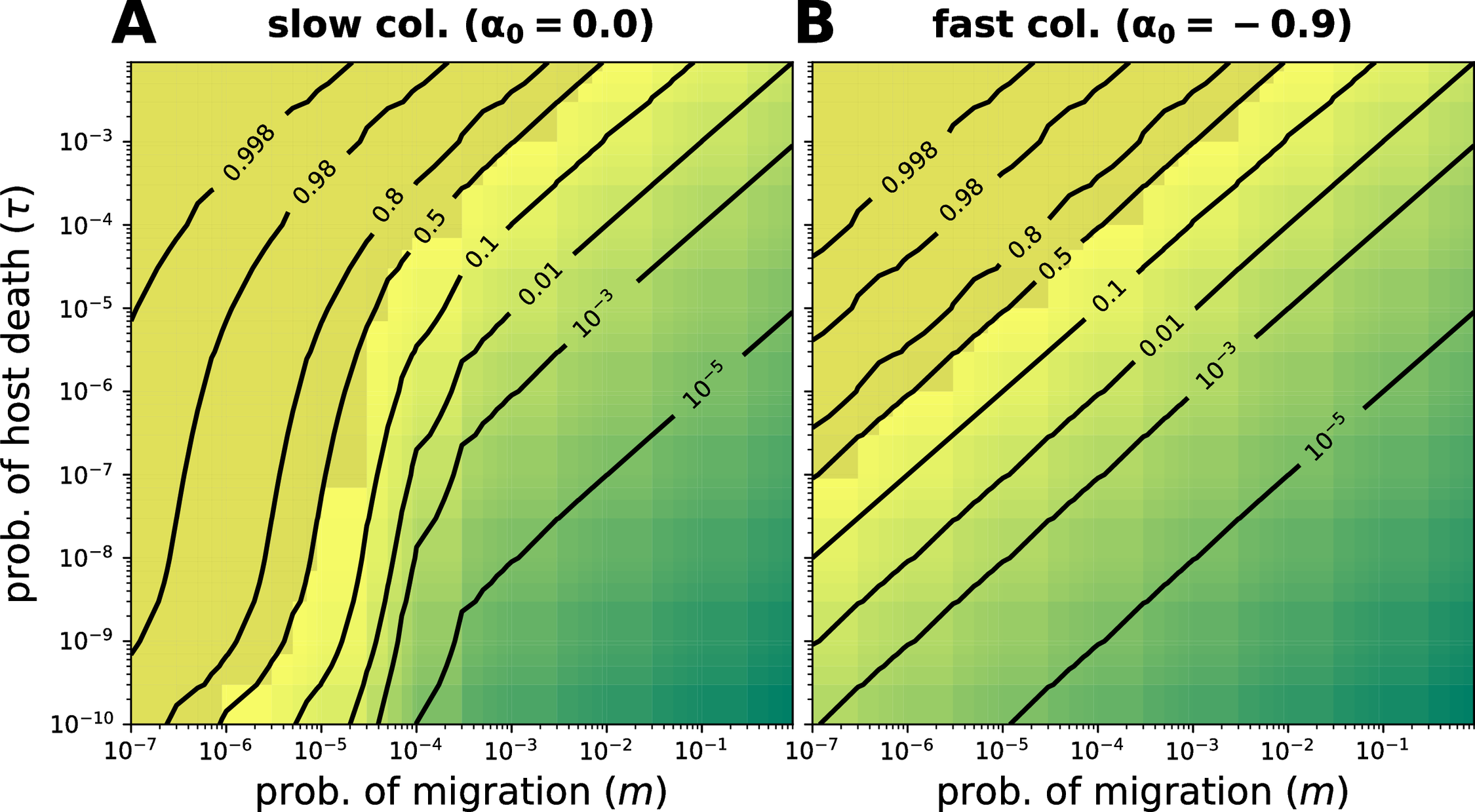

Supplement: S6 Fig — The P[x0 > (N − 1)/N] is shown, Eq (10). (A) Migration (m) is the main driver of the microbe-free state, but still interacting with the probability of host death-birth (τ). The microbe-free state prevails for small m, increasing in the direction of a short host lifespan (large τ). (B) Although a faster occupation of empty space decreases its probability, microbe-free hosts are still expected. Moreover the host lifespan (via τ) becomes more relevant. Other parameters: N = 104. We use Eq (5a) where no definition of pi and αi is required. (TIF) [file pcbi.1008392.s007.tif]

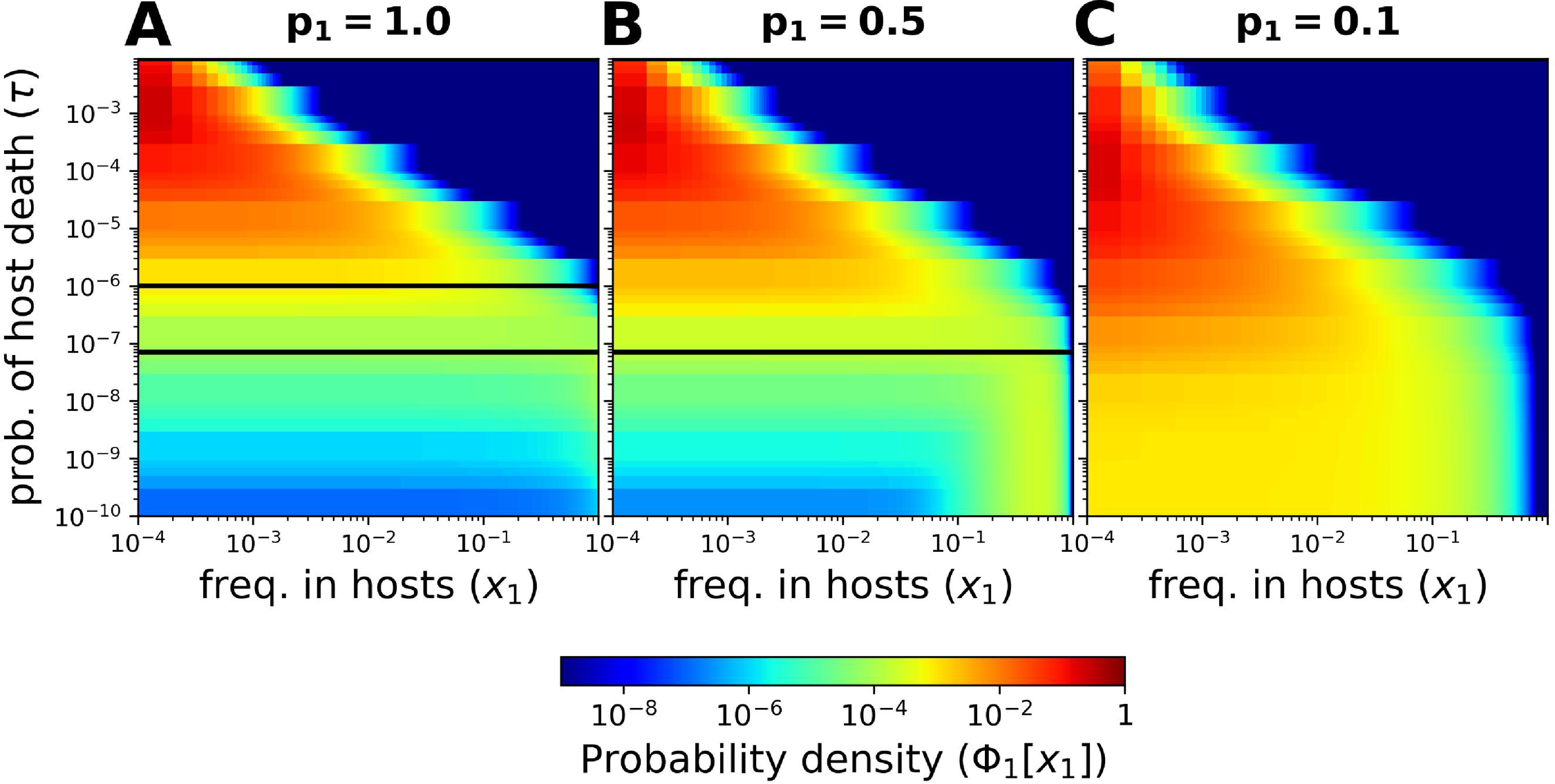

Supplement: S7 Fig — The cross-section of Fig 4 where m = 10−3 is shown. (A). If there are only microbes of type 1 in the pool of colonizers (p1 = 1), small τ implies that there is a single maximum at x1 = 1—the hosts tend to be fully occupied. Bimodality is observed for 7·10-8≲τ≲10-6—some hosts are occupied, but some remain empty. For large τ, hosts tend to remain empty and the distribution has a single maximum at x1 = 0. Black lines indicate the boundaries separating them (see Fig 4). (B) If the microbe is present in the pool of colonizers at p1 = 0.5, no bimodality is observed. For small τ the frequencies are representative of the pool of colonizers and for large τ most hosts do not contain microbe 1. (C) If the microbe is rare in the pool of colonizers, p1 = 0.1, the distribution has a single peak at x1 = 0. This occurs for all values of τ shown here, because there is not enough time in the host to reflect the small number of microbe 1 individuals in the pool of colonizers (any probability smaller than 10−9 was considered as zero, N = 104 and α0 = α1 = 0). (TIF) [file pcbi.1008392.s008.tif]

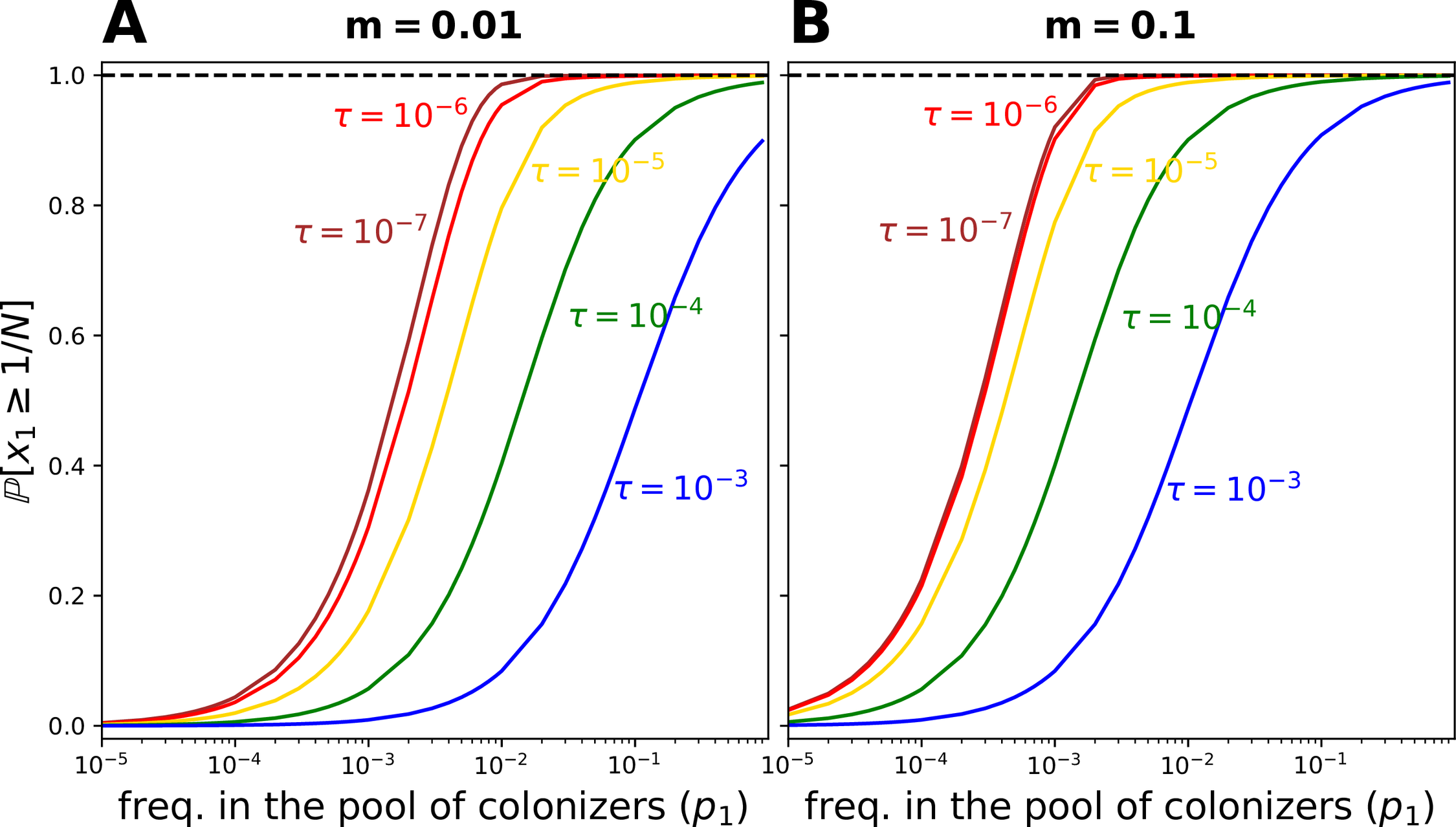

Supplement: S8 Fig — The results of multiple probabilities of host death-birth events (τ) are shown. Overall, the probability of colonization increases with the frequency in the pool of colonizers (p1), but decreases as the host lifespan shortens (larger τ). A smaller migration (m) decreases the probability. Other parameters: N = 104 and α0 = α1 = 0. (TIF) [file pcbi.1008392.s009.tif]
