# Supplementary material for: Stochastic colonization of hosts with a finite lifespan can drive individual host microbes out of equilibrium
Source: PLoS Comput Biol. 2020 Nov 2;16(11):e1008392. doi: 10.1371/journal.pcbi.1008392 (PMC7660904; doi:10.1371/journal.pcbi.1008392)
Supplement: Attachment — Submitted filename: PCOMPBIOL-D-20-00223.pdf [file pcbi.1008392.s010.pdf]

The manuscript introduces a new approach to study host-microbiome symbiosis by incorporating life-span of the host in simple mathematical and computational models. By assuming a neutral population dynamics for the colonized microbes, this model provides a null model for studying the phenomena. The model is simple but exhibits a rich set of outcomes, which, while being not entirely unpredictable, are intriguing and provide useful insights on the importance of host life-span. To the best of my knowledge the model is novel and the same approach has not been used so far to study the effect of finite life-span in symbiotic relationship between host and microbiome. I cannot see any specific potential flaws in the idea, models and interpretation of the results that could cast doubts on the main conclusions. Thus, in my opinion, the manuscript may be published in PLOS Computational Biology after the following and, perhaps, other comments are addressed:

1. As described by the authors previous applications of neutral models to microbiome data have generally ignored demographic processes in the host level such as reproduction and death. However those rare publications that consider host life-cycle on the dynamics, like (Zeng 2018) should be cited. Also holobiont models, although mainly focus on selection, seem to me relevant to be cited in the introduction, where advances and experiences in that field can be useful for audiences of the current manuscript (see for example Roughgarden 2018).
2. Reference 1 (Costello 2012) is not in my opinion an appropriate reference for the claim that "Migration between these individual habitats and ecological drift within them play important roles in structuring these communities." This paper counts diversification and environmental selection beside dispersal and drift the main drivers of community assembly and their focus on drift is less than anything else.
3. Lines 48-49: "To simplify our analysis, we consider a population of hosts that is sufficiently large to track only frequencies." The essential condition for tracking frequencies instead of counts is the large size of the microbiome ($N \gg 1$) which is considered in the lines 52-53. Large number of hosts must be needed to have a sufficiently large ensamble to draw statistical conclusions in the paper for example in the phase diagrams.
4. Caption of Fig. 1: "Every hosts is an identical habitat with ..." is better to be written as "Hosts are identical habitats, each with ...".
5. Fig. 2-B: I couldn't understand how hosts are located over m axis. How the reader must conclude that different hosts are in different regimes

of migration rate? How big is the range of migration rate in nature? The authors just mention that “The parallel lines along the m axis indicate that migration may depend on physiological, environmental and behavioural factors.” Please elaborate on how the points are located and how long the lines are extended in reality.

6. Lines between 60 and 61: For the mathematical model the authors consider one host as an ensemble representative. Without clarification, this assumption (which is correct) is confusing, after the model has been introduced to the reader in line 48 and by figure 1.A with large number of hosts. Please mention this point before starting to solve mathematical model.

7. Lines between 60 and 61: A fitness parameter is introduced for microbes while it is not used in the simulations and no further investigation is done related to this parameter. Please mention in the text that α_i is fixed and equal to 0 in the remaining of the manuscript.

8. Lines between 60 and 61: Model description needs some modifications to make it precise. In the mathematical model population dynamics of microbial taxa of a host is considered. According to equation 1, the subscripts i and j refer to taxa (x_i is the relative abundance or frequency of taxon i). So, sentences like “In each time step, a randomly selected focal microbe i dies” or “it is replaced by a microbe j from the same host” are confusing. An individual of *taxon i* dies and could be replaced by *an offspring* of taxon j. Also these two sentences are wrong from another aspect. The updating rule for each time step, according to equation 1, is selection of one site (not a microbe) that may include also empty space (Eq. 1-c) and it is not replaced necessarily by a microbe but also by empty space (Eq. 1-b). Please rewrite this paragraph more precisely.

9. Related to the previous comment I suggest to avoid the term focal because this term is usually reserved in biology (especially in ecology) for those taxa and species that play an important role in ecosystem function.

10. Lines between 60 and 61: What is the acceptable range of α_0 and α_i?

11. Lines between 60 and 61: The authors explain α_0 as a parameter that “controls how rapidly the unoccupied space within a host is filled with microbes. Another way to interpret this parameter is as the level of resistance the host poses to colonization by microbes.” However α_0 in the model only controls substitution rate of empty spaces with the microbes *inside* the host (internal microbes) and cannot be interpreted

as colonization factor (or colonization probability) in general. Thus, for a fixed m: "For $\alpha_0 > 0$ empty space poses an increased resistance to being colonized **by an internal microbe**, while $\alpha_0 < 0$ increases the probability that unoccupied space is filled with **an internal** microbe". In the extreme case of very large α_0 colonization is only external, i.e. internal microbes have no chance in comparison to empty space to colonize. In this case if the hosts have the chance to reach equilibrium the final frequency of the microbes inside each host is similar to the pool. When internal colonization is stuck (large α_0) the only parameters that matter are m and τ and their ratio $\frac{\tau}{m}$ determines the dynamics. This is the reason behind the (close to) linear contours in Fig. 3A, 4A, 5A. On the other hand for moderate or small values of α_0 the internal dynamics is more relevant and the first colonizers determine the fate of the system. So α_0 controls the relevant speed of internal and external colonization (leading to the results seen in Sec. 3.1) and when α_0 is very small m gets more and more irrelevant while τ and α_0 govern the dynamics (Fig. 3B, 5B).
So what I can conclude is that α_0 is a parameter that controls how important the internal (local) population dynamics is in comparison to external migration. The biological relevance of such a parameter remains as a question, i.e. why the tissue or the microbiome must have resistance against internal microbes but not the environment? This issue must be addressed by the authors and the definition of α_0 as colonization probability must be changed in all instances in the manuscript.

12. Eq. 1: A general reader does not necessarily interpret δ as Kronecker delta. So please, as it is done in the supplementary, point it in the explanation of this equation.

13. Eq. 1: To avoid confusion please introduce p_i right after the equation or preferably in the description of the model (lines between 60 and 61) when it is mentioned that "With probability m, it is replaced by a randomly selected microbe from the pool of colonizers." Also mention that $p_0 = 0$.

14. Lines 61-69: Description of the equation only explains meaning of the equations for microbial taxa. Their interpretation for empty space, x_0, is different, e.g. x_0 does not increase by immigration or reproduction of type 0 within host.

15. Lines 67-69: "In terms of immigration from the environment, all microbes are identical. Only the frequency in the pool of colonizers, pi,

matters." is better to be written as: "Probability of immigration from the environment is proportional to the frequency in the pool of colonizers". See also 13th concern.

16. lines 73-74: $\Phi_i[x_i, t]$ might be perceived as "the change of the probability density of x_i through time t". To avoid this please rewrite this sentence, for example: the time evolution (or dynamics) of probability density of x_i, $\Phi_i[x_i, t]$.
17. Please provide a reference for Fokker-Planck equation. A general reader is not familiar with this equation and its interpretation, like why "$a_i[x_i]$ describes the deterministic part of the change and $b_i^2[x_i]$ describes changes due to randomness."
18. Line 100: The sentence is finished by the equation and line 101 is a new sentence. Related to this comment, please use correct punctuations for all equations. They are part of the sentences.
19. Something that is confusing for me is that the authors first use the large N approximation to have continuous frequencies, which is only relevant for Fokker-Planck equation (neither for the master equation nor the individual-based model). Then they realize that Fokker-Planck equation of their system cannot be solved either analytically or numerically. Although the derivation of this equation is interesting and instructive in general but how is it helping the delivery of the message of the manuscript? See also 34th point.
20. Eq. 7: Which transition matrix in 7.2 is used to solve this equation? Before or after local transition approximation? With or without resetting term? To me the explanation of how the model is solved seems unclear. See also 34th point.
21. Line 111: In the case of finite lifespan what does a stationary state or distribution mean, when in the finite lifespan a variety of transient states coexist (Fig. 2)?
22. Line 148: Figures S1-4, ..., S1-7 are the first figures of the supplementary that are referred in the text. Why are they enumerated from 4?
23. It is expectable that the conclusion of this study be generalized to explain the existence of enterotypes and alternative microbial states in biological studies. Hence, I think it must be discussed that under which circumstances we are eligible to map the results to empirical data. When is a system in short lifespan, fast internal and slow external colonization regime (Fig. 2-B and S1-1-B)?

24. Caption of Fig. 2: "(B) When host**s** are colonized ..."
25. Fig. 4: What is the value for α_0? Why only $\alpha_1 = 0$ is reported here? see point 7. The same for Fig. 6, S1-6, S1-7.
26. What are the values for p_1 and p_2 in Fig. 3, Fig. 5, S1-2, ..., S1-5?
27. Line 240: Isn't it the case that for shorter lifespan we expect to see microbe-free host?
28. Sec 3.4: Although this section has a specific message by focusing on "rapid proliferation of the first colonizer", however the text is basically a lengthy repetition of the lines 206-215 and the captions of Fig. 3 and 4. The reader at that points reads the caption and the short explanation of different regimes. I suggest to make this section shorter with a digest of the main message.
29. The results of the current study and the conclusions will be used in future to understand microbiome communities. Specifically as it is claimed this model explains alternative microbial states observed in empirical studies. However one may ask to which extent the results of a extremely simple model with two taxa are generalizable? The manuscript does not discuss this question that with n taxa how many alternative state are expected? Does this model explain for example how in human gut microbiome with more than 500 taxa we have around 3 distinct enterotypes?
30. I can not see the last four lines of the discussion a logical conclusion of the current manuscript. How we can conclude that ecological drift is fundamental? As it is mentioned by the authors the current model can serve as a null model that can generate some patterns of biological systems. It shows that with a very simplistic neutral model some level of coexistence of alternative states, including the microbe-free state, is possible. With the results of this study how one can reject the importance of selection and ecological interactions?
31. Caption of Fig. S1-1: Are other parameters of the simulations as in Fig 2?
32. Caption of Fig. S1-4...7: What does "the mean of simulations" mean? The average over steady (final) state of 500 host samples according to Eq. 6 or the average over time? What does "the mean of the model" mean?
33. Sec 7.2: The switch between T and T^* in the derivation of Fokker-Planck equation is confusing and need to be explained more clearly in each case. Please explain the local transition approximation and how it

changes T (to T^*). Does it mean that transition rates are only non-zero for $\Delta x \ll 1$? Does it mean that you consider a tridiagonal matrix?

34. Sec 7.2: At the end of this section it is mentioned that numerical solution of the model, Eq. 5, can be found using finite element method while the main text says that the numerical error of its solution is large and inconsistent and so the master equation is used instead. Which approach is finally used to generate plots?

References:

Zeng Q, Rodrigo A. Neutral models of short-term microbiome dynamics with host subpopulation structure and migration limitation. Microbiome. 2018 Dec 1;6(1):80.

Roughgarden J, Gilbert SF, Rosenberg E, Zilber-Rosenberg I, Lloyd EA. Holobionts as units of selection and a model of their population dynamics and evolution. Biological Theory. 2018 Mar 1;13(1):44-65.
