# Supplementary material for: Stochastic colonization of hosts with a finite lifespan can drive individual host microbes out of equilibrium
Source: PLoS Comput Biol. 2020 Nov 2;16(11):e1008392. doi: 10.1371/journal.pcbi.1008392 (PMC7660904; doi:10.1371/journal.pcbi.1008392)
Supplement: Attachment — Submitted filename: reply_to_reviewers.pdf [file pcbi.1008392.s011.pdf]

We thank the reviewers for their detailed and constructive feedback. We believe its implementation now reflects on a clearer delivery of the message, improved readability, and broader discussion. Please find below our point-to-point reply.

Our code is accessible in GitHub (https://github.com/romanzapien/microbiome-hostspan).

Reviewer #1:

The manuscript introduces a new approach to study host-microbiome symbiosis by incorporating life-span of the host in simple mathematical and computational models. By assuming a neutral population dynamics for the colonized microbes, this model provides a null model for studying the phenomena. The model is simple but exhibits a rich set of outcomes, which, while being not entirely unpredictable, are intriguing and provide useful insights on the importance of host life-span. To the best of my knowledge the model is novel and the same approach has not been used so far to study the effect of finite life-span in symbiotic relationship between host and microbiome. I cannot see any specific potential flaws in the idea, models and interpretation of the results that could cast doubts on the main conclusions. Thus, in my opinion, the manuscript may be published in PLOS Computational Biology after the following and, perhaps, other comments are addressed:

1. As described by the authors previous applications of neutral models to microbiome data have generally ignored demographic processes in the host level such as reproduction and death. However those rare publications that consider host life-cycle on the dynamics, like (Zeng 2018) should be cited. Also holobiont models, although mainly focus on selection, seem to me relevant to be cited in the introduction, where advances and experiences in that field can be useful for audiences of the current manuscript (see for example Roughgarden 2018).

Thank you for pointing this out. We apologize for not covering such developments in our introduction. We now mention Zeng et al., 2015, Roughgarden J., 2020 and Van Vliet et al., 2019 in our introduction (lines 25 to 29).

2. Reference 1 (Costello 2012) is not in my opinion an appropriate reference for the claim that “Migration between these individual habitats and ecological drift within them play important roles in structuring these communities.” This paper counts diversification and environmental selection beside dispersal and drift the main drivers of community assembly and their focus on drift is less than anything else.

Agreed. Now we reference instead to Zhou, et al., 2017.

3. Lines 48-49: “To simplify our analysis, we consider a population of hosts that is sufficiently large to track only frequencies.” The essential condition for tracking frequencies instead of counts is the large size of the microbiome ($N \gg 1$) which is considered in the lines 52-53. Large number of hosts must be needed to have a sufficiently large ensamble to draw statistical conclusions in the paper for example in the phase diagrams.

Thank you for the suggestion. We have changed lines 53-54 accordingly.

4. Caption of Fig. 1: “Every hosts is an identical habitat with …” is better to be written as “Hosts are identical habitats, each with …”.

Thank you for the suggestion. We have changed caption of Fig. 1 accordingly.

5. Fig. 2-B: I couldn’t understand how hosts are located over m axis. How the reader must conclude that different hosts are in different regimes of migration rate? How big is the range of migration rate in nature? The authors just mention that “The parallel lines along the m axis indicate that migration may depend on physiological, environmental and behavioural factors.” Please elaborate on how the points are located and how long the lines are extended in reality.

Thank for pointing this out. We apologize for the lack of clarity of Fig 2-B. Its aim was to provide intuition without providing or claiming quantitative detail. We have now modified it to make it simpler and clarifying that it provides only intuition about the importance of the parameters m and τ, and connecting the model to biological examples (sketched hosts). The caption has been simplified accordingly.

Regarding the range of parameters, these could in principle be obtained by inferring m and τ from data, but such a procedure may be somewhat cumbersome: For example, the values of m we obtained in Sieber et al. assume that microbiomes are in equilibrium. Nonetheless, prior knowledge of a host lifestyle can give us a rough idea about the ranges of the parameters: For example, given the short lifespan of *C. elegans* a large τ is expected.

Similarly, the feeding mechanism of *C. elegans* might pose a bottleneck, suggesting a small m. In principle, m can range from 0 (no environmental microbes going in) to 1 (only external migration and no internal reproduction). Our previous studies which attempted to obtain the migration rate m from data for multiple species showed that it can basically span the whole range from almost 0 to almost 1 (Sieber et al. PloS Biol 2019).

6. Lines between 60 and 61: For the mathematical model the authors consider one host as an ensemble representative. Without clarification, this assumption (which is correct) is confusing, after the model has been introduced to the reader in line 48 and by figure 1.A with large number of hosts. Please mention this point before starting to solve mathematical model.

Thank you for the suggestion. We have changed line 66 accordingly. Now we start the paragraph by mentioning the model derivation focuses on the events within a single host.

7. Lines between 60 and 61: A fitness parameter is introduced for microbes while it is not used in the simulations and no further investigation is done related to this parameter. Please mention in the text that α_i is fixed and equal to 0 in the remaining of the manuscript.

Thank you for pointing this out. We apologize for not making this point clear. Now we mentioned that α_i is fixed and equal to 0 after the probabilities of events are introduced (lines 90 to 91). From this point on, α_i is mentioned only where clarification is needed.

8. Lines between 60 and 61: Model description needs some modifications to make it precise. In the mathematical model population dynamics of microbial taxa of a host is considered. According to equation 1, the subscripts i and j refer to taxa (x_i is the relative abundance or frequency of taxon i). So, sentences like “In each time step, a randomly selected focal microbe i dies” or “it is replaced by a microbe j from the same host” are confusing. An individual of taxon i dies and could be replaced by an offspring of taxon j. Also these two sentences are wrong from another aspect. The updating rule for each time step, according to equation 1, is selection of one site (not a microbe) that may include also empty space (Eq. 1-c) and it is not replaced necessarily by a microbe but also by empty space (Eq. 1-b). Please rewrite this paragraph more precisely.

Thank you for pointing this out. We have rewritten this paragraph according to your suggestion (lines 66 to 80). Now the update rule refers to a site, that can be either a microbe or unoccupied space at any given time. And whose replacement can be either a microbe or unoccupied space.

9. Related to the previous comment I suggest to avoid the term focal because this term is usually reserved in biology (especially in ecology) for those taxa and species that play an important role in ecosystem function.

Thank you for pointing out this source of potential confusion. We have removed the word focal from the manuscript.

10. Lines between 60 and 61: What is the acceptable range of α_0 and α_i?

The range of α_0 and α_i goes from -1 to infinity. We have included this specification (lines 78 to 79).

11. Lines between 60 and 61: (11.1) The authors explain α_0 as a parameter that “controls how rapidly the unoccupied space within a host is filled with microbes. Another way to interpret this parameter is as the level of resistance the host poses to colonization by microbes.” However α_0 in the model only controls substitution rate of empty spaces with the microbes inside the host (internal microbes) and cannot be interpreted as colonization factor (or colonization probability) in general. Thus, for a fixed m: “For $\alpha_0 > 0$ empty space poses an increased resistance to being colonized by an internal microbe, while $\alpha_0 < 0$ increases the probability that unoccupied space is filled with an internal microbe”.

Thank you for the suggestion. We have rewritten the relevant paragraph (lines 74 to 78). Now we emphasize that α_0 is only relevant for the reproduction of internal microbes.

(11.2) In the extreme case of very large α_0 colonization is only external, i.e. internal microbes have no chance in comparison to empty space to colonize. In this case if the hosts have the chance to reach equilibrium the final frequency of the microbes inside each host is similar to the pool. When internal colonization is stuck (large α_0)

the only parameters that matter are m and τ and their ratio $\frac{\tau}{m}$ determines the dynamics. This is the reason behind the (close to) linear contours in Fig. 3A, 4A, 5A. On the other hand for moderate or small values of α_0 the internal dynamics is more relevant and the first colonizers determine the fate of the system. So α_0 controls the relevant speed of internal and external colonization (leading to the results seen in Sec. 3.1) and when α_0 is very small m gets more and more irrelevant while τ and α_0 govern the dynamics (Fig. 3B, 5B). So what I can conclude is that α_0 is a parameter that controls how important the internal (local) population dynamics is in comparison to external migration. The biological relevance of such a parameter remains as a question, i.e. why the tissue or the microbiome must have resistance against internal microbes but not the environment? This issue must be addressed by the authors and the definition of α_0 as colonization probability must be changed in all instances in the manuscript.

The relevance of α_0 relies on its role within the death birth-immigration process. While birth requires an internal favorable environment for a microbe to double, immigration does not require this favorable internal environment, instead the new individual originates externally and it is seeded internally. Additionally, if microbes are internalized already, α_0 influences how likely is for them to remain in a host. We distinguish this from (1 - m), that instead determines the overall influence of the internal population on the change of composition.

12. Eq. 1: A general reader does not necessarily interpret δ as Kronecker delta. So please, as it is done in the supplementary, point it in the explanation of this equation.

Thank you for the suggestion. We have included an explanation of δ in lines 83 to 84.

13. Eq. 1: To avoid confusion please introduce p_i right after the equation or preferably in the description of the model (lines between 60 and 61) when it is mentioned that "With probability m, it is replaced by a randomly selected microbe from the pool of colonizers." Also mention that $p_0 = 0$.

Thank you for the suggestion. We now introduce p_i and specify $p_0 = 0$ lines 67 to 69.

14. Lines 61-69: Description of the equation only explains meaning of the equations for microbial taxa. Their interpretation for empty space, x_0, is different, e.g. x_0 does not increase by immigration or reproduction of type 0 within host.

Thank you for pointing this out. We apologize for not stating this distinction. Now we mention the interpretation of the probabilities for a microbial taxon and unoccupied space, explicitly (lines 85 to 89). This also includes equations for the selection and drift terms of the Fokker-Plank approximation - Eq. (3) and Eq. (4).

15. Lines 67-69: "In terms of immigration from the environment, all microbes are identical. Only the frequency in the pool of colonizers, pi, matters." is better to be written as: "Probability of immigration from the environment is proportional to the frequency in the pool of colonizers". See also 13th concern.

Thank you for the suggestion. We have changed lines 67 to 69 accordingly.

16. lines 73-74: $\phi_i[xi;t]$ might be perceived as "the change of the probability density of x_i through time t". To avoid this please rewrite this sentence, for example: the time evolution (or dynamics) of probability density of x_i, $\phi_i[xi;t]$.

Thanks. We have changed lines 94 to 95 accordingly.

17. Please provide a reference for Fokker-Planck equation. A general reader is not familiar with this equation and its interpretation, like why "$a_i[x_i]$ describes the deterministic part of the change and $b_i^2[x_i]$ describes changes due to randomness."

Thank you for pointing this out. Now we include a reference to Gardiner, 2009.

18. Line 100: The sentence is finished by the equation and line 101 is a new sentence. Related to this comment, please use correct punctuations for all equations. They are part of the sentences.

We have now used correct punctuation.

19. Something that is confusing for me is that the authors first use the large N approximation to have continuous frequencies, which is only relevant for Fokker-Planck equation (neither for the master equation nor the individual-based model). Then they realize that Fokker-Planck equation of their system cannot be solved either analytically or numerically. Although the derivation of this equation is interesting and instructive in general but how is it helping the delivery of the message of the manuscript? See also 34th point.

Thank you for pointing this out. We apologize for not providing an argument for such organization of the manuscript. We believe the message is inherent to the stochastic process itself, independent of the approach used to solve it. To produce our figures involving the stationary distribution, we chose the Master Eq. (7), which proved to be numerically very robust. Nonetheless, expressing the process in the form of the Fokker-Planck equation offers several benefits: Most importantly, it provides intuition about the effect of host death. Moreover, it connects to previous literature that assumes $\tau = 0$ (Sloan et al., 2006). It also allows to go beyond the topic of microbiome research and connects to the physics literature that deals with the same underlying principle. Accordingly, we have rewritten the paragraph leading to Eq. (7). Now we state what we believe is gained from thinking about the stochastic process in the shape of the Fokker-Plank approximation.

20. Eq. 7: Which transition matrix in 7.2 is used to solve this equation? Before or after local transition approximation? With or without resetting term? To me the explanation of how the model is solved seems unclear. See also 34th point.

Thank you for pointing this out. We use the transition matrix with probabilities Eq. (1b-c), including the resetting term, Eq. (1a). This is a matrix with main diagonal equal to zero, upper and lower diagonals with probabilities related to the process of death and birth-immigration of microbes, and a column with value τ at either $x_i = 0$ or $x_0 = 1$, related to host death events. Now we make this explicit in the main text after introducing Eq. (7).

21. Line 111: In the case of finite lifespan what does a stationary state or distribution mean, when in the finite lifespan a variety of transient states coexist (Fig. 2)?

We mean the collection of individual transient host states that are stationary at the population level. Now we make this statement previous to introducing Eq. (5).

22. Line 148: Figures S1-4, …, S1-7 are the first figures of the supplementary that are referred in the text. Why are they enumerated from 4?

Thank you for pointing this mistake out. We have changed the supplementary figures order accordingly.

23. It is expectable that the conclusion of this study be generalized to explain the existence of enterotypes and alternative microbial states in biological studies. Hence, I think it must be discussed that under which circumstances we are eligible to map the results to empirical data. When is a system in short lifespan, fast internal and slow external colonization regime (Fig. 2-B and S1-1-B)?

Thank you for the suggestion. Now we address the topic in the discussion in lines 402 to 420.

24. Caption of Fig. 2: “(B) When hosts are colonized ...”

Thank you for pointing this mistake out. We have corrected caption 2.

25. Fig. 4: What is the value for α_0? Why only $\alpha_1 = 0$ is reported here? see point 7. The same for Fig. 6, S1-6, S1-7.

Now we have indicated that $\alpha_0 = 0$ in the relevant captions.

26. What are the values for p1 and p2 in Fig. 3, Fig. 5, S1-2, …, S1-5?

For this figure we use Eq. (5a) where no definitions of p1 and p2 are required. That is because the assumption of neutrality allows putting all taxa together as $(1 - x_i)$ if the focus of analysis is just x_0. We have made this explicit at the end of the captions.

27. Line 240: Isn't it the case that for shorter lifespan we expect to see microbe-free host?

Yes. Fig S1-6 suggests that the microbe-free state will most likely be observed for shorter living hosts. But also, in combination with a low migration from the pool of colonizers. We have rewritten lines 281 to 286 to clarify this.

28. Sec 3.4: Although this section has a specific message by focusing on "rapid proliferation of the first colonizer", however the text is basically a lengthy repetition of the lines 206-215 and the captions of Fig. 3 and 4. The reader at that points reads the caption and the short explanation of different regimes. I suggest to make this section shorter with a digest of the main message.

Thank you for the suggestion. Now we have shortened the section.

29. The results of the current study and the conclusions will be used in future to understand microbiome communities. Specifically as it is claimed this model explains alternative microbial states observed in empirical studies. However one may ask to which extent the results of an extremely simple model with two taxa are generalizable? The manuscript does not discuss this question that with n taxa how many alternative state are expected? Does this model explain for example how in human gut microbiome with more than 500 taxa we have around 3 distinct enterotypes?

Thank you for raising this topic. Now we have addressed this in the discussion in lines 402 to 411.

30. I can not see the last four lines of the discussion a logical conclusion of the current manuscript. How we can conclude that ecological drift is fundamental? As it is mentioned by the authors the current model can serve as a null model that can generate some patterns of biological systems. It shows that with a very simplistic neutral model some level of coexistence of alternative states, including the microbe-free state, is possible. With the results of this study how one can reject the importance of selection and ecological interactions?

Thank you. We indeed cannot reject the importance of selection and ecological interactions. In fact, we do not aim to reject them. Instead, we propose that ecological drift is already sufficient to produce some of the observed patterns, often attributed to selection without justification. Non-neutral processes could act on top of these neutral processes. We have rephrased the last part of the discussion (lines 433 to 439) to reflect these statements more accurately.

31. Caption of Fig. S1-1: Are other parameters of the simulations as in Fig 2?

Thank you for pointing this out. Yes, the same set of parameters apart from *m*. We have now specified this in the caption of Fig. S1-4.

32. Caption of Fig. S1-4…7: What does "the mean of simulations" mean? The average over steady (final) state of 500 host samples according to Eq. 6 or the average over time? What does "the mean of the model" mean?

Thank you for pointing this out. We meant the average over the steady state of 500 host samples according to Eq. (6). We have now modified the captions of Fig. S1-1…3 accordingly.

33. Sec 7.2: (33.1) The switch between T and T* in the derivation of Fokker-Planck equation is confusing and need to be explained more clearly in each case. Please explain the local transition approximation and how it changes T (to T*).

Thank you for pointing this out. We have rewritten the derivation. Specifically, we refer to the role of transitions from neighboring and non-neighboring states in the master equation.

(33.2) Does it mean that transition rates are only non-zero for $\Delta x \ll 1$?

No, we approximate the non-zero transition rates using a Taylor expansion.

(33.3) Does it mean that you consider a tridiagonal matrix?

Not exactly. In the transition matrix, the main diagonal equals zero. The upper and lower diagonals represent transitions to neighboring states. Non-neighboring transitions, produced by host death events, are given by a column of τ, at the state $x_i = 0$ for microbes or $x_0 = 1$ for unoccupied space. Now, we have made this explicit in the main text after introducing Eq. (7).

34. Sec 7.2: At the end of this section it is mentioned that numerical solution of the model, Eq. 5, can be found using finite element method while the main text says that the numerical error of its solution is large and inconsistent and so the master equation is used instead. Which approach is finally used to generate plots?

We have used the master equation, Eq. (7), to produce the results and figures shown. Now we make an argument for such decision in the main text, before Eq. (7) is introduced.

Reviewer #2:

In this manuscript, the authors present an expanded (near) neutral model for microbial community assembly in hosts of varying lifespans, to explain how patterns of microbial colonization and co-existence can emerge from ecological drift when the timescale of the host is considered. Using a combination of model analytics and simulations, the authors present a set of interesting insights into the microbial community assembly process within a host, particularly in clarifying the relationship between lifespan and migration in determining convergence to the infinite-lifespan case. This manuscript makes a convincing case for considering microbiomes as an assembly process rather than an equilibrium scenario, and the model presented will provide a useful starting point for considering further expansions into the near-neutral scenario where interactions among microbes are permitted to vary.

(1) It would be interesting if the authors were to speculate further on the possible uses and expansions of this model. For example, in the Discussion, priority effects are mentioned but not expanded upon. The model makes predictions about what the distribution of colonization intensities/frequency of unoccupied individuals should look like for an ordinary Markov process on a population of identical hosts – expansions could be used to make predictions for departures from the current model in any of these assumptions. Some of the interesting cases might involve the question of memory (e.g. priority effects). For example, microbial “death” might be bursty (as seems to be the case in zebrafish) – can this still be a Markov process (maybe a Levy walk?), or is it necessary to allow the system to remember prior states? How can we tell these possibilities apart? These expansions are beyond the scope of the current work, but increasing the future work component of the Discussion along the lines of this or other biologically relevant scenarios would improve the accessibility of the manuscript.

Thank you for the suggestion. Now, we have addressed these topics in the Discussion in lines 421 to 432. Specifically, having microbiome transfer from parents to offspring, changes in the dynamics originating from the microbiome or host, and the possible ‘bursty’ microbial death in nature.

Minor points

(2) Define p_i in text before presentation of equation 1.

We apologize for the missing definition. We have now defined p_i before Eq. (1).

(3) 4D-F - It would be informative to plot out (as a supplement) the heatmap of x_i values that correspond to these plots.

Thank you for the suggestion. Now we include a supplementary figure (S1 Fig 7.) showing the underlying distributions in Fig. 4D-F.

References:

Zeng Q, Rodrigo A. Neutral models of short-term microbiome dynamics with host subpopulation structure and migration limitation. Microbiome. 2018 Dec 1;6(1):80.

Roughgarden J, Gilbert SF, Rosenberg E, Zilber-Rosenberg I, Lloyd EA. Holobionts as units of selection and a model of their population dynamics and evolution. Biological Theory. 2018 Mar 1;13(1):44-65.

Roughgarden J. (2020) Holobiont Evolution: Mathematical Model with Vertical Vs. Horizontal Microbiome Transmission. *Philosophy, Theory, and Practice in Biology*, 12(2).

Sieber, M., Pita, L., Weiland-Bräuer, N., Dirksen, P., Wang, J., Mortzfeld, B., ... & Hentschel, U. (2019). Neutrality in the Metaorganism. PLoS biology, 17(6), e3000298.

Sloan, W. T., Lunn, M., Woodcock, S., Head, I. M., Nee, S., & Curtis, T. P. (2006). Quantifying the roles of immigration and chance in shaping prokaryote community structure. *Environmental microbiology*, 8(4), 732-740.

Van Vliet, S., & Doebeli, M. (2019). The role of multilevel selection in host microbiome evolution. *Proceedings of the National Academy of Sciences*, *116*(41), 20591-20597.

Zeng, Q., Sukumaran, J., Wu, S., & Rodrigo, A. (2015). Neutral models of microbiome evolution. *PLoS computational biology*, *11*(7).

Zhou, J., & Ning, D. (2017). Stochastic community assembly: does it matter in microbial ecology?. *Microbiology and Molecular Biology Reviews*, 81(4).
