# Supplementary material for: Stochastic colonization of hosts with a finite lifespan can drive individual host microbes out of equilibrium
Source: PLoS Comput Biol. 2020 Nov 2;16(11):e1008392. doi: 10.1371/journal.pcbi.1008392 (PMC7660904; doi:10.1371/journal.pcbi.1008392)
Supplement: Attachment — Submitted filename: Review-revisedversion.pdf [file pcbi.1008392.s012.pdf]

The authors did a very good job in revising their paper. Almost all of my concerns and those of the other reviewer have been addressed satisfactorily, just a few things that are left,

1. The dynamics of empty space is not explained clearly and in some cases is not correct. According to the equations (1b) and (1c) empty space is treated the same as other sites.
   Based on the presented interpretation of these equations in the manuscript,
   - the dynamics in (1b) is described as a random selection of the target site (all except i) which may be replaced by the offspring of a randomly selected source site (i) and
   - the dynamics in (1c) as a random selection of the target site (i) which may be replaced by the offspring of randomly selected source site (all except i).

   The point is that when i is a target in (1c), how we can interpret its population reduction via encountering empty space as "inability to reproduce" (line 88)? Why inability to reproduce should decrease the population? It keeps the population unchanged. In my opinion here theoretical reproduction of an empty space means death of the target site. However it is not clear why the death rate of a taxa must depend on the abundance of empty space.
   Related to this point the sentence "or it is not replaced with probability proportional to $(1 + \alpha_0)x_0$" (line 71-72) is not correct. It is in fact replaced by empty space according to (1c). The probability of no replacement is given by (1d).
   One important aspect of these equations is that selection of the source is proportional to its frequency. Is it clear for the authors why abundance of empty space must promote its existence? For a taxa we expect to have more offsprings by having more individuals but why it should be true for empty space as well, even in the case of $\alpha_0 = 0$? Isn't it the case, especially in neutral model, that in a normal situation existence of empty space in a habitat means existence of unoccupied resources and easier reproduction of the extant individuals?
   I think this aspect of the model must be addressed in the manuscript to avoid extension of its conclusions to all cases.

Minor:

1. It is just a matter of taste but adding the explanation about the estimation of τ and m, provided in the response, to the manuscript could

be instructive for the reader.

2. In the revised version the usage of i and j is sloppy and confusing. In the equations (1) you use i while in the following explanation of the equations i and j are mixed! In the equations (2)-(5) you use j but in their explanations i is used! After equation 5 everything is explained with i.

3. Lines 108-110: I would write these lines as "For $\tau \to 0$, the last terms in Eq (2) vanish, recovering the usual Fokker-Planck equation of the neutral model without host death [3], while for $\tau > 0$ these additional terms describe the change due to host death, where a new, microbe-free host appears." because the word "additional" is in comparison to "the usual Fokker-Planck equation of the neutral model" which is referred in the second sentence.

4. Again as a matter of taste I would rearrange the lines from 116 to 157. In the current version you
   (a) describe that you are going to solve Eq 5.
   (b) explain the alternative interpretation of those equations in lines 128-133.
   (c) explain the benefits of Eq 5 in lines 133-137.
   (d) start to solve them.
   Isn't it better and less confusing to have something like c-b-a-d?
